# Physician-Modified Custom Made Endograft Adding an Outer Branch to Correct a Fenestration Offset

**DOI:** 10.1177/15266028251384208

**Published:** 2025-11-05

**Authors:** Lorenzo Torri, Petroula Nana, José I. Torrealba, Tilo Kölbel, Giuseppe Panuccio

**Affiliations:** 1 German Aortic Center, Department of Vascular Medicine, University Heart and Vascular Centre UKE Hamburg, Hamburg, Germany

**Keywords:** physician-modified endograft, endovascular aortic repair, symptomatic abdominal aortic aneurysm, ruptured abdominal aortic aneurysm

## Abstract

**Purpose:**

To describe the use of a physician-modified endograft (PMEG) through the incorporation of an outer branch within a fenestrated custom-made device (CMD) originally designed for another patient with similar anatomy.

**Technique:**

An 80-year-old male, with a symptomatic 80 mm juxtarenal aneurysm, was managed urgently using a readily available fenestrated CMD, designed for a different patient. While 3 of the 4 fenestrations were appropriately aligned with the intended target vessels (TVs), the fourth fenestration, targeting the left renal artery, was incorporated 15 mm lower than the vessel’s orifice. To overcome this offset, a 6 mm covered stent (Viabahn, Gore & Associates, Flagstaff, Arizona) was latero-terminal sutured to the fenestration, creating a retrograde outer branch. The procedure was successful, followed by an uneventful postoperative course. Predischarge computed tomography confirmed the patency of the TV and the freedom from endoleak.

**Conclusion:**

Urgent setting may demand further modifications of readily available CMD, including the incorporation of branches in fenestrated stent grafts, in order to accommodate in patient’s anatomy. Future standardization of the modification techniques may further expand their utilization.

**Clinical Impact:**

This case introduces a physician-modified endograft technique incorporating an outer branch into pre-existing fenestrated custom-made device (CMD) for urgent complex aortic aneurysm repair. Using an available CMD instead of creating a new PMEG reduces preparation time and procedural complexity while maintaining technical success and target vessel patency. This practical, repoducible approach broadens the applicability of fenestrated technology in urgent settings lacking suitable off-the-shelf options.

## Introduction

Complex endovascular aortic repair with fenestrated or branched endografts (f/bEVAR) has been successfully applied under elective settings.^1,2^ However, custom-made devices (CMDs) require a production period up to 2 months that can lead to adverse events during the waiting period. Along these lines, urgent and emergent cases are usually managed either with commercially available off-the-shelf devices or physician-modified endografts (PMEGs), depending on patient’s anatomy and availability of materials. Off-the-shelf branched devices, as mainly designed for thoracoabdominal aneurysm repair, require extensive thoracic aortic coverage, which may increase the risk of spinal cord ischemia, while their applicability in selected patients, as females, is low as 22%.^2^ The CMDs initially designed for other patients have been selectively used under urgent setting in patients with similar anatomy and provided encouraging early outcomes.^3–7^ However, due to the significant variability in the anatomy of the reno-visceral aortic segment, a fenestrated off-the-shelf device has not been commercially introduced yet, and graft modification by the operator has been suggested in an urgent setting.^3,6^

Herein, we present a novel graft modification technique to adapt a readily available fenestrated CMD to patient’s anatomy in urgent setting.

A written consent has been assigned by the patient, confirming agreement to the publication of the case details and images.

## Case Presentation

**Figure 1.**
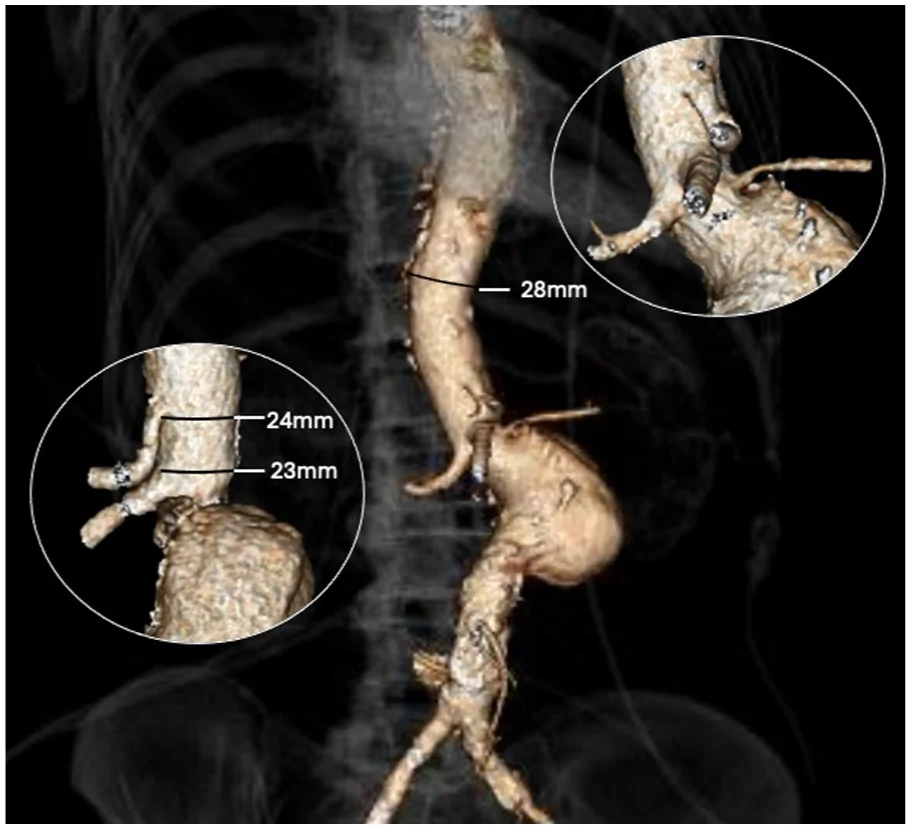
Preoperative 3-dimensional volume rendering computed tomography angiography (3D VR CTA) of Juxtarenal Abdominal Aortic Aneurysm (JAAA). Within circles, the close-up of visceral vessels; intravessel diameter (IVD) at the level of proximal fixation, CT, and SMA.

An 80-year-old man with a history of hypertension and severe aortic valve stenosis presented with abdominal pain, exacerbated by deep palpation. A computed tomography angiography (CTA) revealed an 80 mm juxtarenal aortic aneurysm, without signs of rupture (Figure 1). Considering the patient’s high-risk profile for an open repair of the aortic valve and juxtarenal aneurysm, an urgent simultaneous transcatheter aortic valve implantation (TAVI) and endovascular aortic repair (EVAR) was decided.

**Figure 2.**
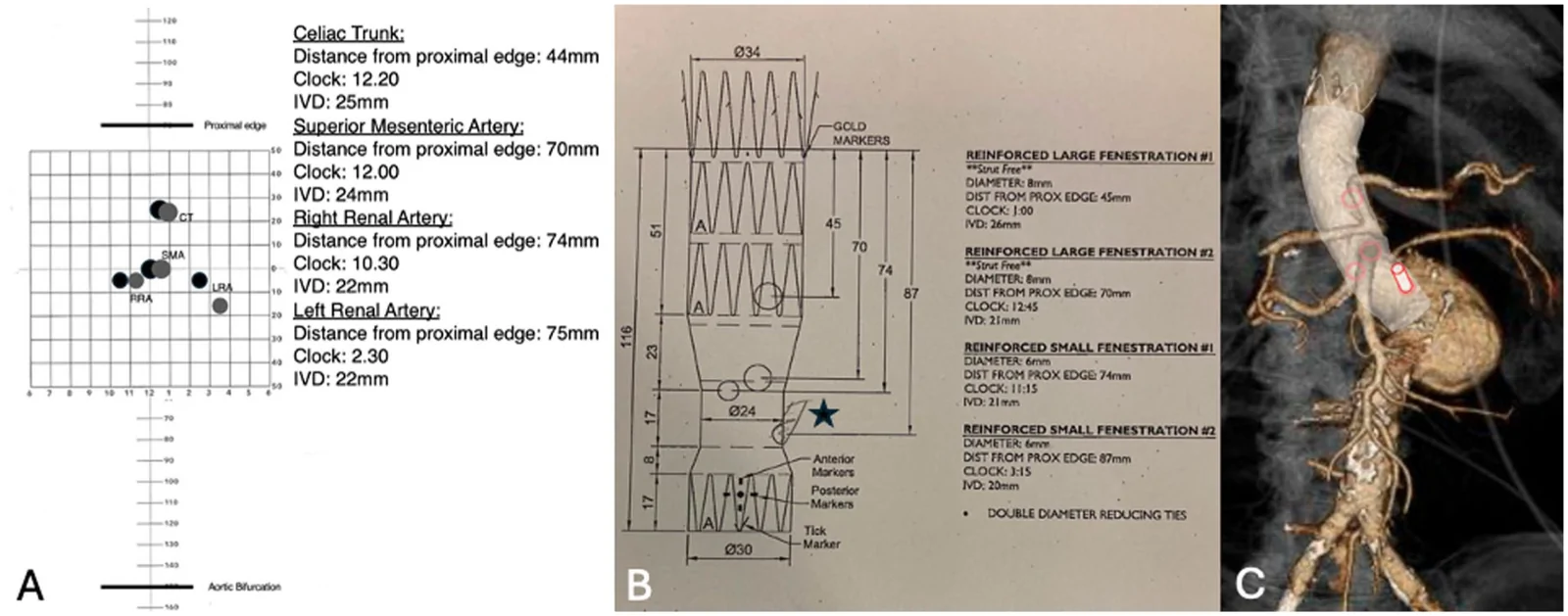
Comparison of patient’s anatomy with the available fenestrated custom-made endograft (CMD fEVAR) plan. (A) Patient’s anatomical plot (black circles) superimposed on the CMD fenestrations (gray circles), detailed vessel information including distance from the proximal edge, clock position, and intravascular diameter (IVD); (B) CMD fEVAR plan highlighting the intended outer branch with a pencil sketch (star); (C) preoperative 3D VR CTA, indicating the positions of CMD fenestrations (red circles) and a 3D representation of the outer branch (red cylinder), illustrating the shape and design of the endograft.

Planning was performed using a dedicated software (Aquarius Intuition, TERARECON Inc, San Mateo, California, Figure 2). As no infrarenal sealing zone was available and given the proximity of the renal arteries to the superior mesenteric artery (SMA), a double parallel graft (chimney) repair was omitted. Given the size of the visceral aorta (23 mm) and to reduce the extension of aortic coverage, the use of the off-the-shelf branched device was also excluded. Considering the availability of stock CMD which could conform otherwise to the patient’s anatomy and the reduced time and complexity to modify such a device compared to a standard off-the-shelf one, a CMD over a fully on-table PMEG was chosen, accepting the higher cost associated. In addition, the small aortic diameter and the extreme aortic angulation at the visceral segment would make the use of an in situ PMEG even more challenging and was omitted. After matching the sizing of the aortic neck with the specifications of the CMD grafts available in our inventory, an endograft was identified (Fenestrated Thoracoabdominal Graft, Cook Medical, Bloomington, Indiana). This graft had a proximal diameter of 34 mm and two 8 mm fenestrations for the celiac trunk (CT) and SMA, and two 6 mm fenestrations for the renal arteries (Figure 2). The oversizing of 21% with sealing 44 mm above the CT (28 mm diameter at this level) was deemed acceptable; for the distal sealing, an extension was possible using an off-the-shelf graft.

The relative distances of the target vessels (TVs) were almost fitting the fenestration positions of the available graft plan, and with slight counterclockwise graft rotation, 3 of 4 fenestrations could be aligned with the TVs, while 1 was located 15 mm below the left renal artery (LRA). To overcome this limitation, a back table modification was planned to create a retrograde outer branch (Figure 2).

### Preparation of the Stent Graft

**Figure 3.**
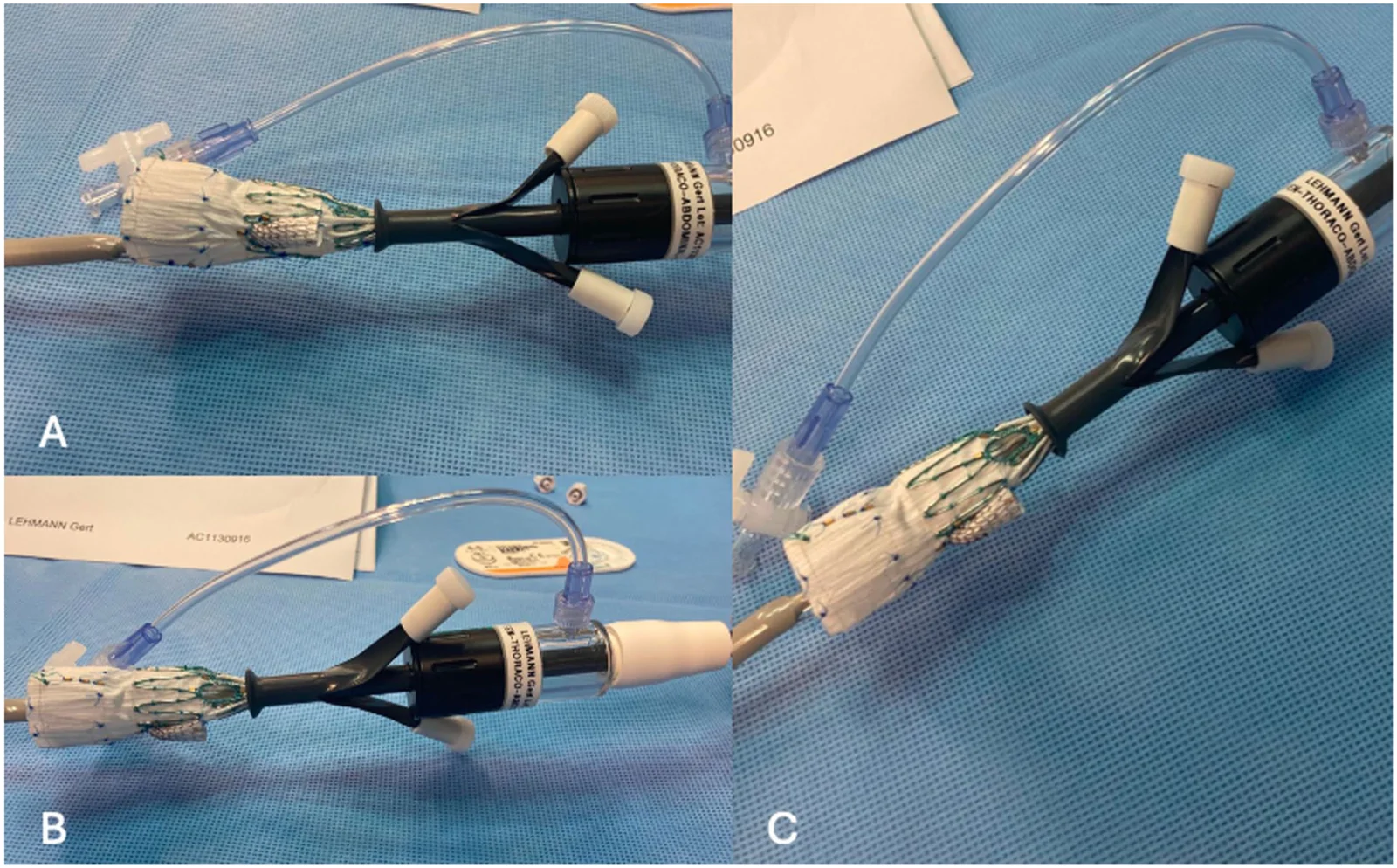
Back table modification. (A), (B), and (C) present various projections of the fenestrated endovascular aneurysm repair (fEVAR) modification. These images illustrate the creation of an outer branch through a latero-terminal anastomosis.

On the back table, the endograft was partially deployed until the fenestration of the LRA was revealed. A 6 × 50 mm Viabahn (W.L. Gore, Flagstaff, Arizona) was deployed and shortened to 15 mm with an oblique cut. The modified stent was then sewed to the fenestration in latero-terminal fashion, using a 4/0 braided suture (Prolene 4/0, ETHICON Johnson & Johnson, New Brunswick, New Jersey), creating a retrograde outer branch, orientated upward and anterior according to TV’s clock position (2:45). The distal part of the neo-branch was fixed to the endograft with a single suture to ensure its stability (Figure 3). Finally, the endograft was re-sheathed and then flushed with carbon dioxide and saline solution;^8^ these preparation required approximately 10 minutes.

## Procedure

The operation was performed in a hybrid room with a fixed imaging system, under fusion guidance. The patient was in a supine position under general anesthesia. Bilateral percutaneous common femoral access was gained under ultrasonographic guidance using Prostar closure devices (Abbott, Lake Forest, Illinois). Systemic heparinization at 100 IU/kg with a target activated clotting time >250 seconds was obtained. During the endograft’s modification, the TAVI procedure was successfully performed.

**Figure 4.**
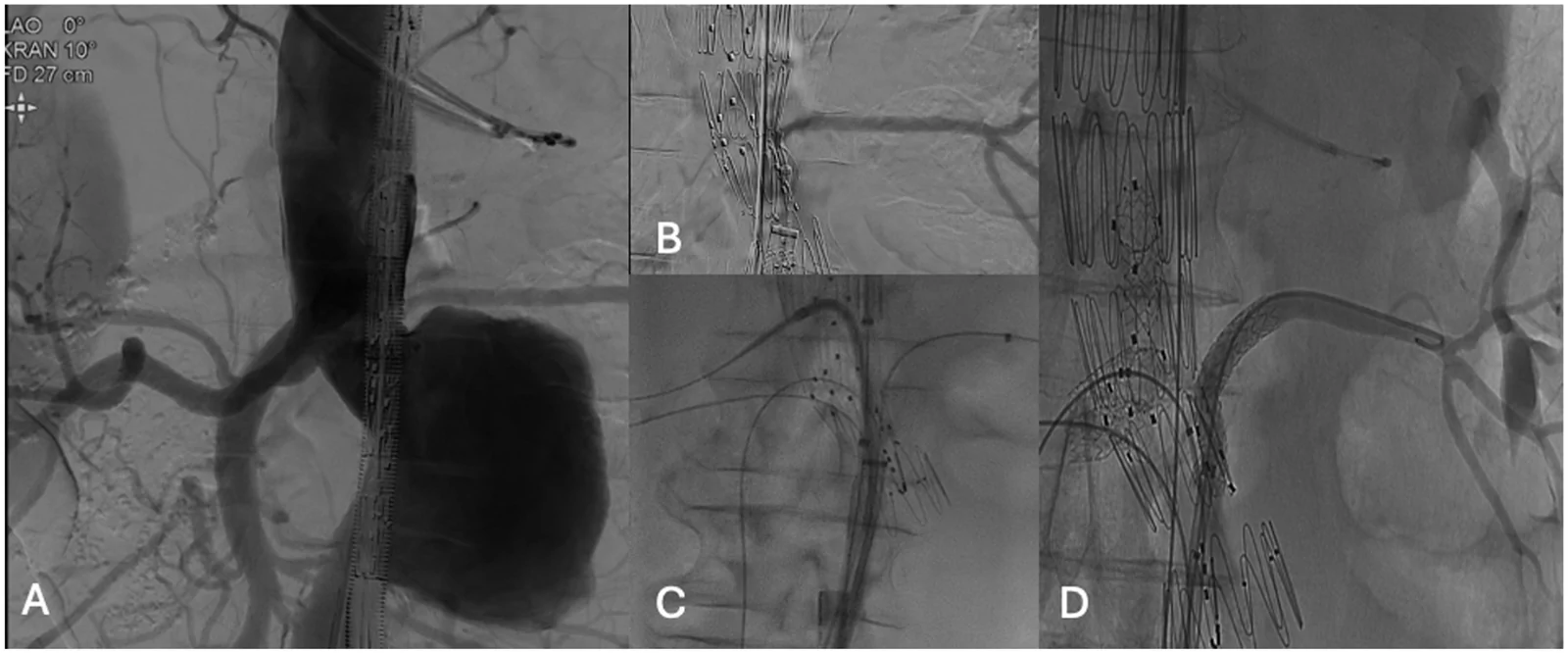
Intraoperative steps. (A) Digital subtraction angiography (DSA) performed prior to the deployment of the CMD fEVAR; (B) cannulation of the outer branch and selective DSA of the left renal artery (LRA); (C) fluoroscopic image displaying the cannulation of all target vessels; (D) selective DSA of the LRA following stenting and flaring.

Afterwards, the CMD-PMEG was advanced from the right side using a 20-French (Fr) sheath (Flexor, Cook Medical) and deployed as per standard fashion. From the left access, a 18Fr sheath was placed. Initially, the branch was catheterized using a Bernstein catheter (Cordis Corporation, Hialeah, Florida) and a hydrophilic .035’ wire (Terumo Medical Corporation, Somerset, New Jersey). A 7Fr Flexor sheath was advanced into the branch to secure it during the next steps. A 7Fr Aptus Tourguide (Medtronic, Dublin, Ireland) was then simultaneously introduced, and the remaining TVs were sequentially catheterized.^9^ The deployment of the CMD-PMEG was completed, followed by molding with a compliant balloon (CODA, Cook Medical). Balloon-expandable covered stents were used to bridge the TVs, CT: 8 x 27 mm iCover (iVascular, Barcelona, Spain), SMA: 9 × 32 mm Advanta V12, and Right Renal Artery (RRA): 7 × 22 mm Advanta V12 (Getinge/Atrium Medical Corporation, Merrimack, New Hampshire). All bridging stents were appropriately flared using a 10 mm balloon. The LRA was bridged last, using a 6 × 38 mm and a 6 × 22 mm Advanta V12. A postdilation using a 7 mm balloon was performed. To achieve distal sealing, the CMD-PMEG was extended using 2 aortic cuffs (30 × 80 and 30 × 58 mm Cook TBE, Figure 4), achieving 40 mm in distal sealing at the level of inferior mesenteric artery (IMA). Due to significant angulation of the infrarenal aorta, an additional relining of the distal aortic extensions was performed using a Giant Palmaz (Cordis, Santa Clara, California).

The completion angiography confirmed the exclusion of the aneurysm and the perfusion of all 4 TVs. Sheaths and wires were retrieved, and complete closure of the access was achieved with Prostar. The duration of the procedure was 270 minutes (of which 60 minutes for the TAVI procedure), with a total fluoroscopy time of 116 minutes and a radiation dose at 23 400 Gy.cm^2^.

**Figure 5.**
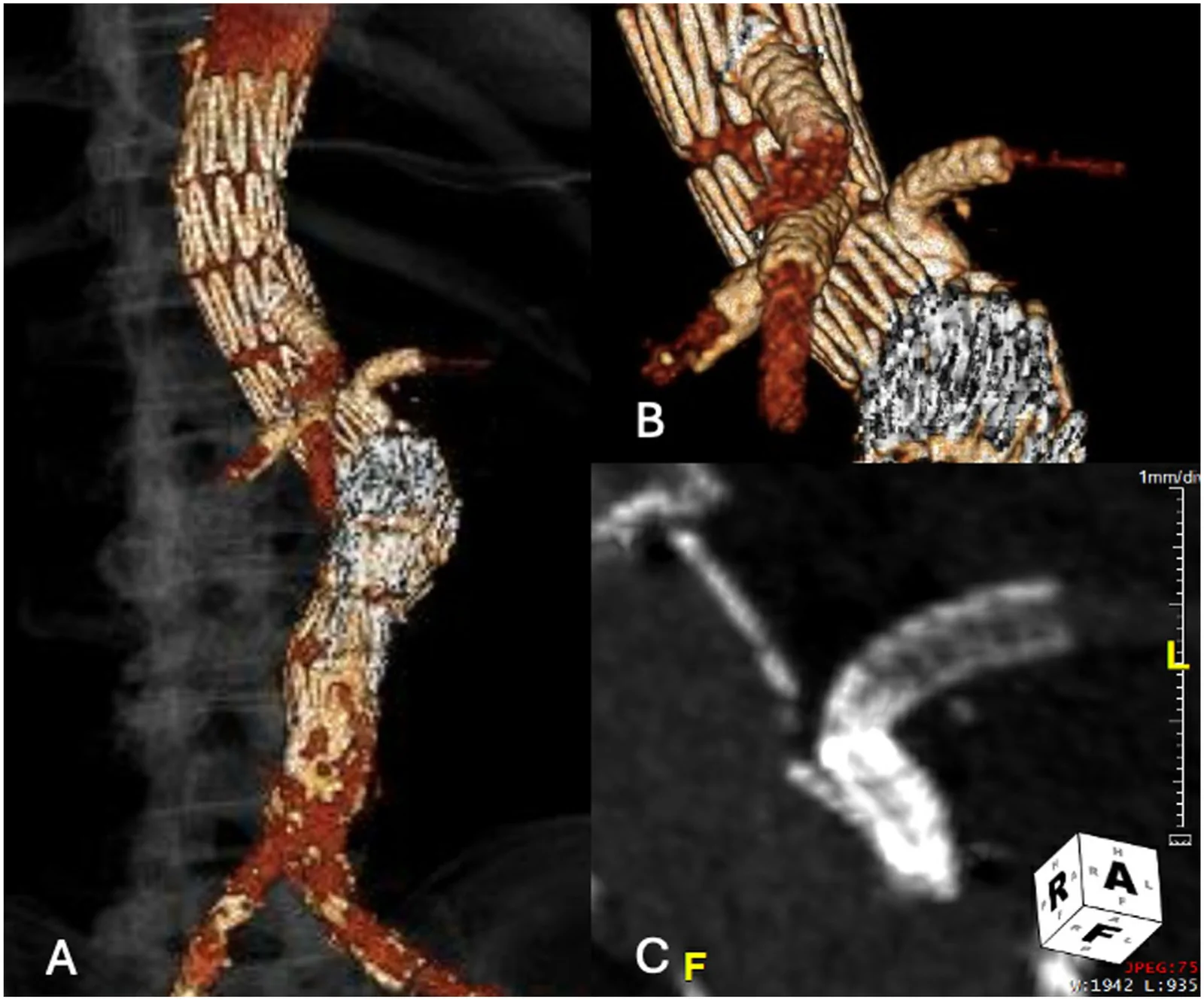
Postoperative CTA. (A) 3D VR of the postoperative CTA demonstrating complete exclusion of the aneurysm, absence of endoleaks, and patency of all bridging stents; (B) close-up view of the visceral vessels as shown in (A); (C) Maximum Intensity Projection (MIP) on Multi-Planar Reconstruction (MPR) illustrating the configuration of the outer branch.

The patient had an uneventful postoperative course. The predischarge CTA showed no endoleak and confirmed the findings of the intraoperative angiography (Figure 5). The patient was discharged on the fifth postoperative day with the addition of aspirin to his home medication regimen; the patient’s preoperative creatinine level was within the normal range at 1.21 mg/dL, a slight postoperative increase was observed, reaching 1.36 mg/dL, which was consistent with the procedure; creatinine levels decreased at discharge to 1.28 mg/dL and returned to normal during follow-up, measuring 1.18 mg/dL at 3 months postoperatively. The patient underwent a 3-month CTA, which was comparable to the predischarge scan. Given the absence of endoleak, the patency of the TVs, and the stability of the aneurysmal sac, a follow-up CTA was scheduled at 9 months.

## Discussion

This technical note introduces an additional tool to expand the use of PMEG in complex aortic aneurysms needing urgent repair. In urgent setting where the available off-the-shelf endovascular solutions are not applicable and open surgery is prohibitive due to patients’ high-risk profile, PMEG may be used with high technical success, of 98%, and a highly acceptable reintervention rate of 7% at 1-year follow-up.^10^

Previously published experience with the t-branch device (Cook Medical) showed encouraging outcomes in urgent and emergent setting.^4^ However, anatomic restrictions, related to the proximal landing zone and visceral aortic diameter, may hamper its use in juxtarenal aneurysms with smaller aortic lumens, as in our case,^2^ where the use of an off-the-shelf device would not permit an easy device rotation, appropriate graft deployment, and consequent TV’s catheterization. Another problem encountered with the off-the-shelf endografts is the need to cover a long segment of the thoracic aorta with the consequent occlusion of segmental arteries and a higher risk of spinal cord ischemia (SCI). The PMEG may be used to overcome these pitfalls, in narrow aortas, as in this case, where the supra-CT aortic coverage was limited to 44 mm.

Up to now several different off-label ways have been described to perform PMEG, using different platforms and reloading systems.^3,6^ To further extend the application of the technique, companies are striving to develop standardized processes to achieve on-label use, providing validated measurements, modification protocols, and reloading kits. However, despite these efforts, two factors remain limiting the wider use of PMEG: the time required for sizing and modification and the unknown long-term graft stability.

Similar to Tsilimparis et al,^6^ the proposed solution based on prefenestrated CMD significantly accelerates the modification process in PMEG. In fact, the presence of diameter-reducing ties, which permit partial graft deployment, and the pre-existing fenestrations expedite further the needed modification process. Compared to the method involving covering and creating new fenestrations, suturing an outer branch appears, in our opinion, even faster. The solution described in this technical report requires only a minor modification, with the branch being fixed to the main body, using a simple Prolene stitch over the preattached nitinol ring, without extended main endograft cuts and stitching on the Dacron material.

Shehab et al^10^ published a case report of a patient managed using a single-SMA fenestration device in addition to 2 in-situ renal fenestrations and supported that the minimal mesenteric ischemia time may be beneficial in these cases. To our opinion, a device that could at least provide a reinforced fenestration for the SMA (precatheterized or not) with no or 1 additional reinforced fenestration for 1 renal artery would permit to decrease significantly both the modification and ischemia times, which may be crucial especially in urgent cases. The addition of another fenestration or branch would not be similarly time consuming and probably would affect less the perioperative outcomes and long-term durability.

Regarding graft stability, the COOK platform has demonstrated robust data over the years and remains the most commonly used in various published experiences. The presence of nitinol rings and precise sutures minimizes the risk of type III endoleak (6.5% perioperative, 1.4% over 2 years, based on recent data from the USA group). However, this advantage could be compromised if the creation of a new fenestration would be required, as suggested by Tsilimparis et al.^6^ Despite the favorable early results, the incorporation of a retrograde outer branch directed at a 90° angle in a setting of marked aortic angulation, as seen in this patient, is not without potential drawbacks. This configuration may theoretically predispose to increased mechanical stress at the bridging stent junction, risking kinking, crushing, or thrombosis over time. These concerns are well-grounded and require close mid- and long-term surveillance. Nonetheless, in our specific case, the short bridging segment, the controlled upward-anterior orientation of the outer branch (clockwise direction 2:45), and the preoperative planning aiming at minimizing torsion on the stent connection were all tailored to mitigate such risks. Furthermore, the bridging stent was flared and reinforced using balloon-expandable covered stents. Moreover, the lower fenestration, positioned 15 mm below the RRA fenestration, offers the option to cover it with a cuff in the event of future complications related to material fatigue. Moreover, the lower fenestration, positioned 15 mm below the RRA fenestration, offers the option to cover it with a cuff in the event of future complications related to material fatigue.

The proposed solution underscores the value of PMEG in urgent settings by introducing an innovative approach with several potential advantages. However, it is important to acknowledge existing limitations, such as the time required for modifications and long-term graft stability. While prefenestrated platforms with diameter-reducing ties present a promising option for standardizing the modification process, ongoing careful assessment of potential risks, including type III endoleak and complications related to material fatigue, is essential. Future studies could provide clarity on the long-term benefits versus risks, particularly given anatomical variability and the specific needs of each patient. Balancing innovation with safety will be crucial to expanding the acceptance and use of this technique in clinical practice while ensuring optimal outcomes for patients.^3–7^

## Conclusion

Urgent setting may demand further modifications of readily available CMD, including the incorporation of branches in fenestrated stent grafts, in order to accommodate in patient’s anatomy. Future standardization of the modification techniques may further expand their utilization.
